# Rosai-Dorfman disease presenting as stridor and hoarseness in a young female patient

**DOI:** 10.1016/j.radcr.2022.11.018

**Published:** 2022-11-28

**Authors:** Vivianne Kokje, Claudio De Vito, Flavia Costa Varela, Yan Monnier, Nicolas Dulguerov, Minerva Becker, Maxime Mermod

**Affiliations:** a Department of Otorhinolaryngology—Head and Neck Surgery, Geneva University Hospitals, 4 rue Gabrielle-Perret-Gentil, CH-1211, Geneva, Switzerland; b Division of Clinical Pathology, Diagnostic Department, Geneva University Hospitals, 4 rue Gabrielle-Perret-Gentil, CH-1211, Geneva, Switzerland; c Service of Radiology, Geneva University Hospitals, 4 rue Gabrielle-Perret-Gentil, CH-1211, Geneva, Switzerland

**Keywords:** Rosai-Dorfman disease, Larynx, Head and neck, Magnetic resonance imaging

## Abstract

Rosai-Dorfman disease is a non-Langherans cell histiocytosis typically revealed by lymphadenopathy. Extranodal involvement occurs in 43% and most commonly involves the head and neck, skin, and bones. Few reports have described laryngeal lesions. We report the case of a Rosai-Dorfman disease in a 27-year-old female, presenting as an obstructing transglottic mass. We provide the results of the MRI and PET-scanner examination. As the treatment relies on surgical excision and the diagnosis depends on pathological examination, we also detail the analysis results that followed the surgical resection. This report highlights the necessity to consider Rosai-Dorfman disease as a potential diagnosis in case of a laryngeal submucosal mass.

## Introduction

Rosai-Dorfman disease (RDD) is a rare non-Langherans cell histiocytosis typically revealed by lymphadenopathy, mainly cervical, in adolescents and young adults. Extranodal involvement occurs in 43% of the patients [1]. Laryngeal involvement is sporadic, with only 13 cases reported in the literature [2]. We report a case of mixed RDD with extranodal transglottic and lymph node involvement with a particular focus on radiological features.

## Case presentation

**Fig. 1:**
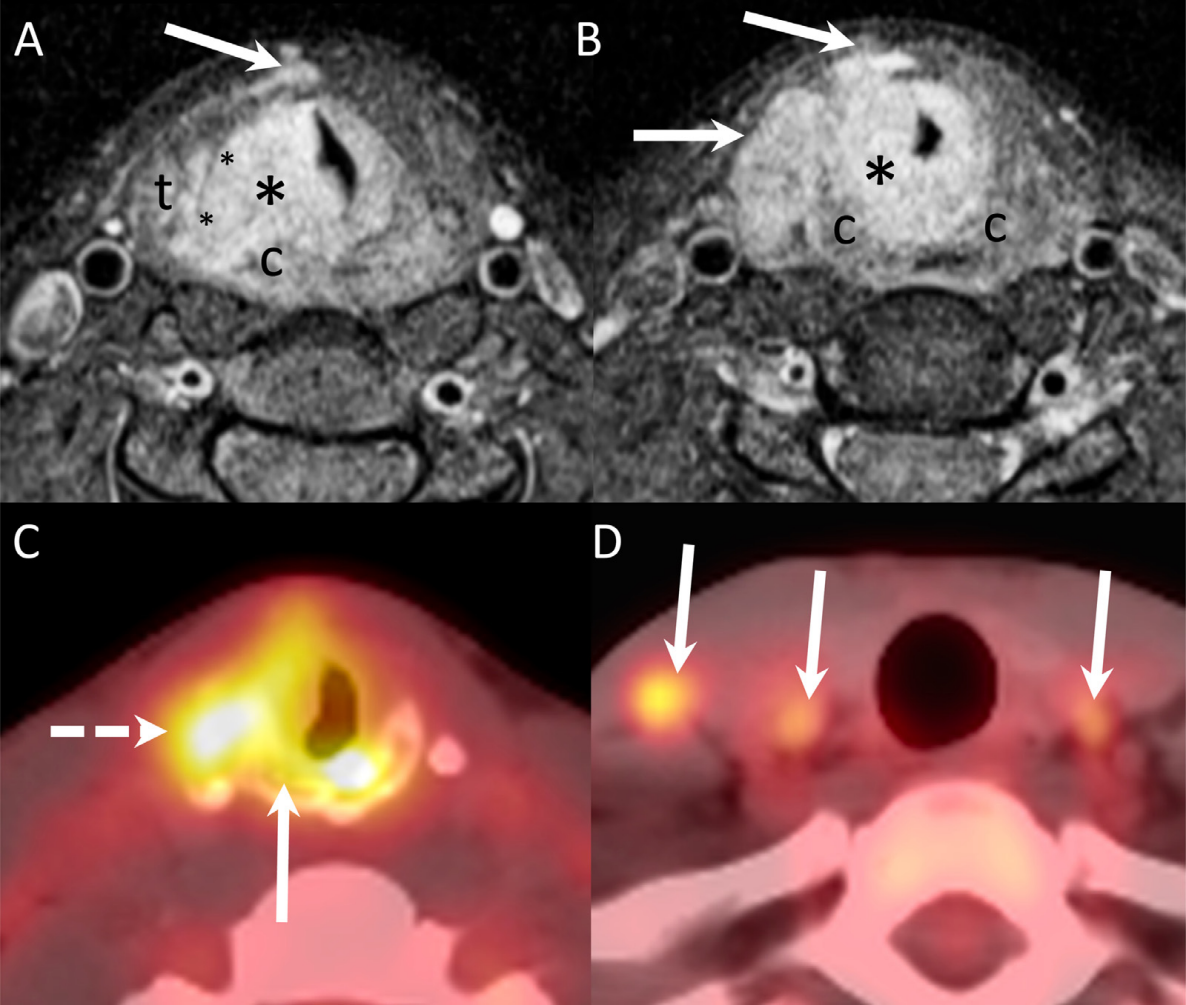
Endoscopic findings (A) direct laryngoscopy before tumor debulking showing right obstructive transglottic submucosal mass; (B) direct laryngoscopy before tumor debulking with Tritube in place; (C) restored airway caliber after laser endoscopic debulking with false vocal cord retractor; D) restored airway caliber after debulking.

A 27-year-old female patient with no significant medical history was evaluated in the hospital for a 2-month history of progressive dyspnea accompanied by biphasic stridor and hoarseness. In-house otorhinolaryngology consultation identified significant hoarseness and a biphasic (expiratory and inspiratory) stridor. Flexible laryngoscopy showed considerable swelling of the right false vocal cord extending to the subglottis, reducing airway caliber by 90% with immobility of the right vocal cord. MRI of the larynx with intravenous contrast revealed a 2 cm × 1.8 cm × 3 cm (latero-lateral × antero-posterior × cranio-caudal) right-sided transglottic submucosal mass lesion invading the right paraglottic space, the cricoid and thyroid cartilage, extending into the soft tissues of the neck and with two ipsilateral enlarged level IV lymph nodes (Fig. 1). Diffusion-weighted imaging revealed restricted diffusivity with an apparent diffusion coefficient (ADC) of 0.7-0.8 × 10-3 mm^2^/s. Based on the morphologic and functional MRI features, the diagnosis of a non-Hodgkin lymphoma was suggested. FDG-PET/CT showed an increased glucose metabolism of the laryngeal mass with a maximum standardized uptake value of 9.7, as well as 3 abnormal lymph nodes (2 level IV nodes on the right and one level IV node on the left) equally suggesting a malignant lesion (Fig. 1). Due to the worrisome findings of airway obstruction and suspicion of malignancy, the patient consented to direct laryngoscopy. She was taken to the operating room for direct suspension laryngoscopy and debulking of the mass to obtain enough tissue for histopathology and restore airway passage.

**Fig. 2:**
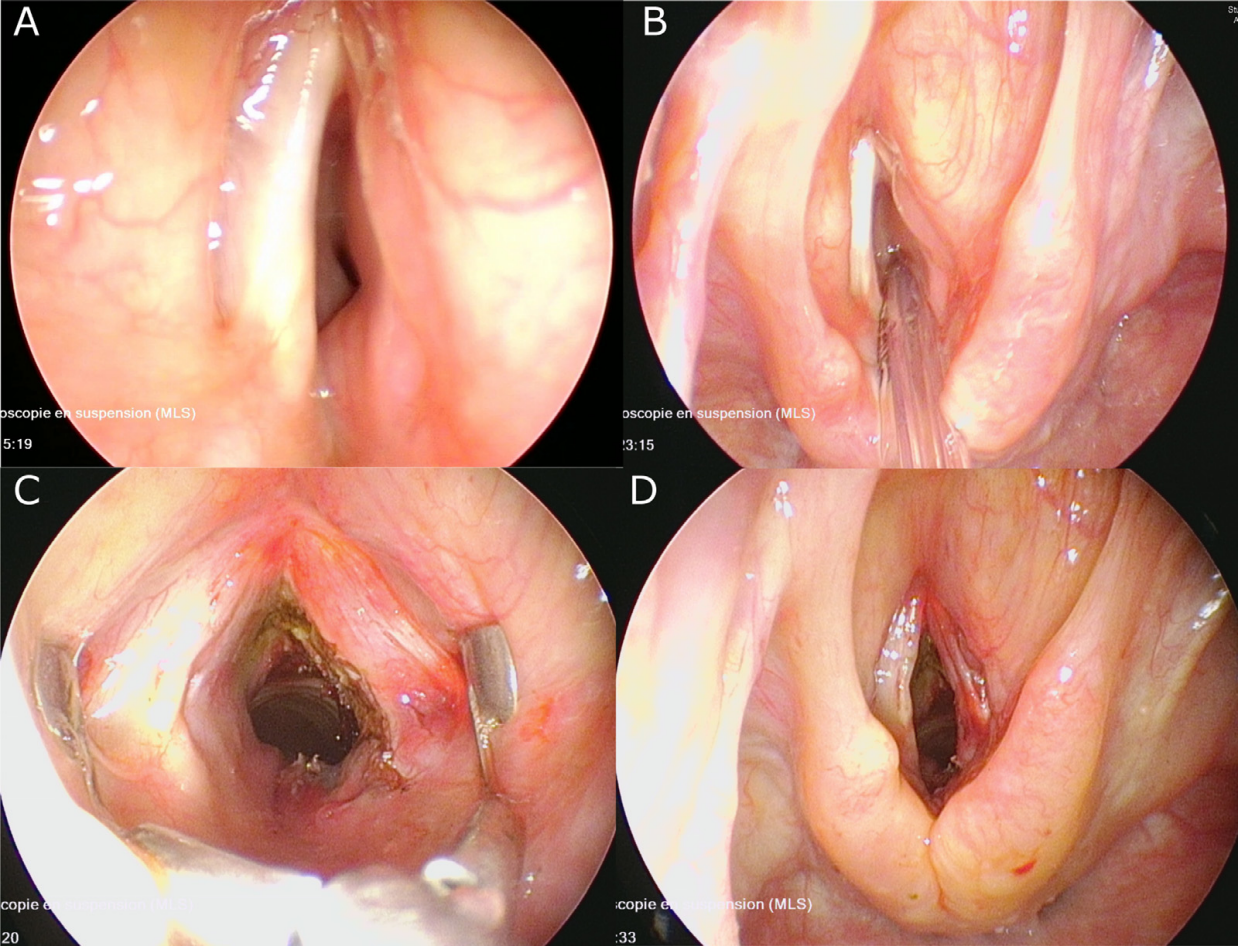
MRI and PET/CT characteristics of the transglottic laryngeal mass with lymphadenopathy. (A) Axial contrast-enhanced fat-saturated T1-weighted image at the glottic level shows a diffusely infiltrating mass (large asterisk), with right paraglottic space invasion (small asterisks), invasion of the thyroid (t) and cricoid (c) cartilage. Arrow points at anterior extra-laryngeal spread. (B) Contrast-enhanced fat-saturated T1-weighted image obtained at the subglottic level shows extensive spread of the lesion (asterisk) outside the larynx into the soft tissues of the neck (arrows). Cricoid cartilage (c). Note massive laryngeal airway narrowing at both levels. FDG-PET/CT images (C and D) show that the transglottic laryngeal lesion with cartilage invasion (arrows) is strongly FDG-avid (maximum standardized uptake value = 9.7). Three suspicious lymph nodes (dashed arrows) were equally identified. The two lymph nodes on the right were later removed to obtain histopathology.

**Fig. 3:**
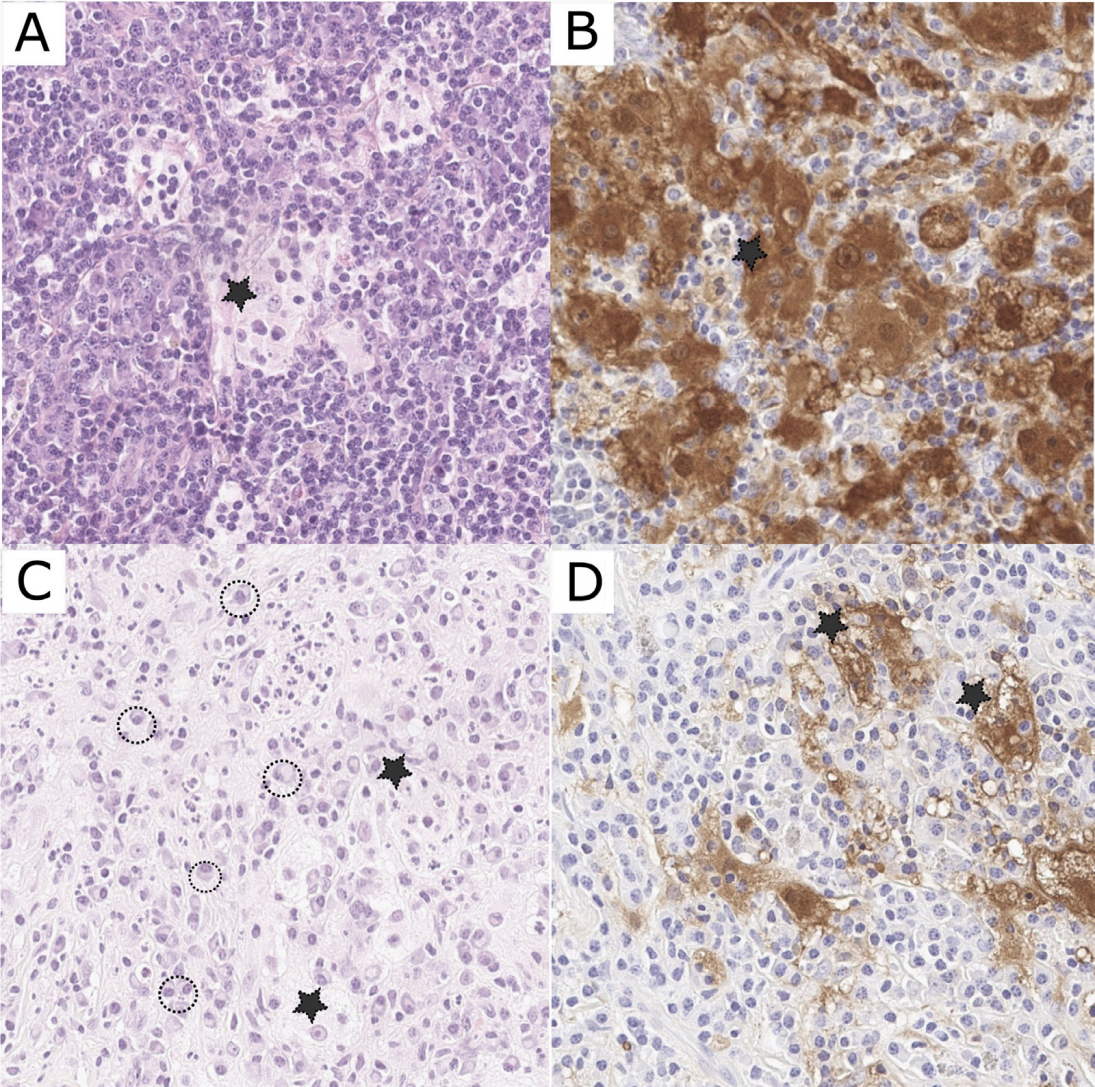
Pathological findings (A) Hematoxylin and eosin (H&E) stained section (×200) showing a pathological lymph node with histiocytic infiltration with emperipolesis (star) in sinusoids. (B) S-100 stained section (×400) of a lymph node showing S-100 positive histiocytes with emperipolesis (star). (C) Hematoxylin and eosin (H&E) and (D) S-100 stained section (×400) of the extralaryngeal mass, showing mixed inflammatory infiltrate (dashed circle) and histiocytic infiltrate with emperipolesis (star).

Direct laryngoscopy demonstrated a submucosal transglottic lesion involving the right side of the larynx, narrowing the laryngeal lumen to less than 0.5 cm (lateral) × 1 cm (anteroposterior) in the infraglottic and subglottic region (Fig. 2). We performed endoscopic laser debulking of the lesion to obtain enough tissue for pathological analysis and restore the airway passage. An incision was made at the right false vocal cord level, allowing for the restoration of an acceptable airway circumference up to the level of the subglottis (Fig. 2). The patient's airway symptoms improved after the procedure and she was discharged from the hospital on postoperative day 4. The histology report revealed a mixed inflammatory infiltration composed mostly of lymphocytes, plasmocytes, neutrophils, and fibrosis. Histological and immunohistochemistry ruled out lymphoma, carcinoma, or sarcoma, but without a definitive diagnosis. To yield a definitive diagnosis, the patient was consented and taken to the operating room for right neck lymph node excision and biopsy of the extralaryngeal tumor component (Fig. 3). The histological analysis of the extralaryngeal mass revealed histiocytes co-expressingCD68 and S100 protein and containing engulfed intact inflammatory cells, a phenomenon referred to as emperipolesis, associated with fibrosis and a prominent inflammatory infiltrate comprised of plasma cells, lymphocytes, and neutrophils. Two lymph nodes were removed and shown also within the lymph node sinusoid S100 positive histiocytes with emperipolesis. BRAF V600E immunohistochemistry was negative. The patient did not receive any other adjuvant treatment. At 6-month follow-up, flexible laryngoscopy showed no signs of recurrence and identified only mild hoarseness as a residual symptom of the disease.

## Discussion

RDD, initially described by Destombe [3] and further characterized as a distinct entity by Rosai-Dorfman [4], is a benign but progressive lymphoproliferative and histiocytic disorder of unknown etiology with good outcome in most cases [5]. Although it is most frequently seen in children and young adults, onset has been reported up to age 74 [6]. The prevalence is about 1:200,000. The pathogenesis of the disease is unclear and likely multifactorial, but an association with immune disease suggests that immune dysregulation may play a role in pathogenesis. A subset of cells can harbor MAPK/ERK pathway alterations, suggesting pathogenesis similar to Langerhans cell histiocytosis and Erdheim-Chester disease [7].

Differential diagnosis between laryngeal RDD and a malignant tumor is challenging as clinical, and imaging features can often mimic the latter. Biopsy often fails to obtain a definite diagnosis. In our case, the diagnosis was made only after a lymph node excision and extralaryngeal tumor biopsy. Histological examination showed an infiltrative mass with numerous histiocytes, with immunohistochemical positivity for S100 and multiple lymphophagocytosis images (ie, emperipolesis). The common differential diagnosis includes infectious lesions, granulomatous lesions, reactive lymphoid hyperplasia with sinus histiocytes (RLHSH), Langerhans cell histiocytosis (LCH), hemophagocytic syndrome, and malignant lymphoma [8,9]. While lymph node involvement is common [10], skin is the most common extranodal site. Other frequent extranodal sites include bone, upper respiratory tract (sinus), the central nervous system, including dura and parenchymal lesions, orbit, and retroperitoneum [11]. Clinical manifestations vary due to the involvement of different organs or systems.

The most common manifestations of laryngeal RDD are dyspnea, stridor, and dysphonia. Other symptoms include cough, dysphonia, and foreign body sensation. The imaging characteristics of laryngeal RDD are not pathognomonic but rather suggest malignancy as the lesions typically display an infiltrative and destructive pattern at CT, PET/CT, and MRI. On MRI, they present hypointensity on T1-weighted images, slight hyperintensity on T2-weighted images and a strong and relatively homogenous contrast enhancement [12,13]. Likewise, cartilage destruction and spread into the soft tissues of the neck accompanied by lymph node involvement as in this case further support the presumptive diagnosis of a malignant condition. On diffusion-weighted imaging, RDD lesions have restricted diffusivity with low ADC values similar to the ADC values of lymphoma (ie, in the range of 0.6-0.8 × 10-3 mm^2^/s). Such low ADC value reflects hypercellularity and densely packed cells. On PET-CT, the lesions are FDG- avid, typically displaying high standardized uptake value values due to the infiltrative and inflammatory process and some authors have suggested that FDG PET/CT could be used to monitor treatment results in systemic RDD [14]. Currently, no clear consensus is available on the treatment strategies. Around 20% of the cases show spontaneous regression without specific intervention, while 70% require particular treatment that may differ according to the involved organ. Treatment includes surgery, steroids, radiation, and chemotherapy. Surgery remains the most effective option for a solitary lesion. In the current case, no further treatment than surgical resection was necessary.

## Conclusion

In conclusion, we report a case of laryngeal RDD with airway compromise, with good outcome following surgical treatment. The underlying pathogenesis, clinical presentation, radiological features, pathology finding, treatment, and results are discussed. We expect this article to help alert clinicians to this condition.

## Patient consent

Informed consent was obtained from the patient for the publication of this case report.
